# Correction of Asymmetric Bowtie Corneal Astigmatism with a Toric Intraocular Lens: Outcomes and Accuracy of Measurement Modes

**DOI:** 10.3390/jpm13030401

**Published:** 2023-02-24

**Authors:** Hao Li, Wenwen He, Donglin Guo, Yanwen Fang, Jiaqi Meng, Keke Zhang, Xiangjia Zhu, Yi Lu

**Affiliations:** 1 Eye Institute and Department of Ophthalmology, Eye & ENT Hospital, Fudan University, Shanghai 200031, China; 2 NHC Key Laboratory of Myopia (Fudan University); Key Laboratory of Myopia, Chinese Academy of Medical Sciences, Shanghai 200031, China; 3 Shanghai Key Laboratory of Visual Impairment and Restoration, Shanghai 200031, China

**Keywords:** toric IOL, asymmetric bowtie astigmatism, cataract

## Abstract

The outcomes of toric intraocular lens (IOL) implantation in correcting asymmetric bowtie corneal astigmatism remain uncertain. The accurate measurement of corneal astigmatism is essential for surgical planning. In this prospective cohort study, patients with asymmetric or symmetric bowtie corneal astigmatism who underwent toric IOL implantation were recruited. Preoperative corneal astigmatism was measured with an IOLMaster and Pentacam (including the simulated keratometry (SimK), total corneal refractive power (TCRP), and wavefront aberration (WFA) modes). At 3 months after surgery, the refractive outcomes and residual astigmatic refractive errors were compared with patients with symmetric bowtie astigmatism. The prediction errors (the differences between the calculated actual corneal astigmatism and the measured corneal astigmatism) were compared among the different measurement modes in the asymmetric group. There were no differences in residual astigmatism between the asymmetric and symmetric groups. However, the mean absolute residual astigmatic refractive error was greater in the asymmetric group than in the symmetric group (0.72 ± 0.42 D vs. 0.53 ± 0.24 D, *p* = 0.043). In the asymmetric group, the mean absolute prediction errors for the IOLMaster, SimK, TCRP and WFA modes were 0.53 ± 0.40, 0.56 ± 0.47, 0.68 ± 0.52, and 0.43 ± 0.40 D, respectively. The Pentacam WFA mode was the most accurate mode (*p* < 0.05). The absolute prediction error of the WFA mode was positively correlated with the total corneal irregular astigmatism higher-order aberrations and coma (r = 0.416 and r = 0.473, respectively; both *p* < 0.05). Our study suggests toric IOL implantation effectively corrected asymmetric bowtie corneal astigmatism. The Pentacam WFA mode may be the most accurate measurement mode, although its accuracy decreased as asymmetry increased.

## 1. Introduction

In recent years, cataract surgery has evolved from a sight-restoring procedure to a refractive procedure. Epidemiological studies have shown that 15–29% of cataract patients have more than 1.5 diopters (D) of corneal astigmatism before surgery [1,2], which cannot be corrected with a conventional monofocal intraocular lens (IOL). Therefore, toric IOLs are usually used in these patients [3,4] and their effectiveness is now well established in regular and symmetric astigmatic eyes [4,5,6].

Contrary to regular astigmatism, irregular astigmatism is another type of astigmatism and defined as an astigmatic state that could not be corrected by a sphero-cylindrical lens. The prevalence of irregular astigmatism is about 40% [7,8]. According to computerized topographic systems, irregular astigmatism can be further classified into different types. Asymmetric bowtie astigmatism is an important one and characterized by unequal slopes of the hemimeridians along a single meridian [9]. The treatment of asymmetric bowtie corneal astigmatism remains challenging for cataract surgeons, and relevant studies are sparse. A previous investigation showed that the residual astigmatism after toric IOL implantation could be as high as 3.0 D in eyes with asymmetric bowtie or other irregular corneal astigmatisms [10]. The difficulty of correcting these astigmatisms lies in the development of a proper surgical plan to correct asymmetric bowtie astigmatism with a symmetric toric IOL.

Measurement accuracy is essential for the surgical plan of patients with corneal astigmatisms. Inaccurate measurement may result in significant residual astigmatism after surgery, which can significantly affect postoperative visual acuity. A study showed that postoperative refractive astigmatism largely affects postoperative visual acuity; 1 diopter left in postoperative refractive astigmatism resulted in 1.5 lines of reduction in postoperative visual acuity at a distance [11]. In recent years, biometric instruments, such as IOLMaster and Pentacam, have become widely used to measure corneal astigmatism. IOLMaster uses telecentric keratometry to reduce the disruption to image acquisition caused by patient movements [12] and usually calculates the corneal power from the anterior surface [13]. Pentacam reconstructs the shape of the anterior and posterior corneal surfaces using Scheimpflug photography principles [14]. The simulated keratometry (SimK) mode (which measures the anterior corneal keratometric power), total corneal refractive power (TCRP) mode (which analyzes the overall refractive status of the cornea, including the anterior and posterior surfaces), and wavefront aberration (WFA, which is the deviation of a reflected wave from a reference unaberrated wave) mode are the three Pentacam modalities that are most commonly used to evaluate corneal astigmatism [15]. However, using different measure methods of the devices lead to significant variability in the values of results. Previous studies showed that using TCRP when concerning the effect of posterior corneal astigmastism was more accurate than SimK to calculate the toric IOL power in eyes with symmetric corneal astigmatism [16,17]. Another study showed that IOLMaster measured significantly greater keratometry readings in the steep axis for all participants. The keratometry and WTW measurements of the IOLMaster and Pentacam cannot be used interchangeably in keratoconic patients [18]. However, it is unclear which measurement mode is most accurate in the surgical planning of toric IOL implantation in eyes with asymmetric bowtie corneal astigmatism.

Therefore, in the present study, we first evaluated the refractive outcomes of toric IOL implantation in eyes with asymmetric bowtie astigmatism, and then compared the prediction errors between four different measurement modes: IOLMaster, SimK, TCRP, and WFA of Pentacam.

## 2. Materials and Methods

### 2.1. Patients

Patients who underwent cataract surgery with toric IOL implantation (AT Torbi 709M IOL, Carl Zeiss AG, Oberkochen, Germany) between November 2018 and March 2020 at the Eye and ENT Hospital of Fudan University were continuously recruited for this study. The symmetry of corneal bowtie in all patients was evaluated with a Pentacam HR (Oculus Inc., Wetzlar, Germany). Based on the corneal topography, the inferior–superior index (I–S) was defined as the corneal curvature of the inferior points minus the corneal curvature of the superior points on a steep axis at 5 mm on the sagittal curvature (front) image, whereas the superior–inferior index (S–I) was defined conversely. The skewed radial axis index (SRAX) was defined as the axis difference between the two lobes of the bowtie [13]. Asymmetric bowtie corneal astigmatism was defined as I–S > 1.5 D, S–I > 2.5 D, or SRAX > 22° [19]. Only patients with an I–S > 1.5 D or an S–I > 2.5 D were enrolled in our study. Patients with corneal pathologies, such as keratoconus, pterygium or corneal scars, contact lens wear within the preceding 2 weeks, glaucoma, strabismus, previous trauma or ocular surgery, zonular weakness, severe fundus pathology, and uveitis, were excluded. Patients who experienced intraoperative or postoperative complications, such as posterior capsular rupture, severe and persistent corneal edema, pupillary capture of the IOL, or misalignment of the toric IOL by more than 10°, those with postoperative visual acuity less than 20/63, and those who were lost to follow-up, were also excluded from the analyses. Finally, 30 eyes in the asymmetric group and 30 eyes matched with age, gender and corneal astigmatism magnitude in the symmetric groups were analyzed. The flow chart is shown in Figure 1.

### 2.2. Preoperative Examinations

The routine preoperative examinations included the assessment of visual acuity, a slit-lamp examination, fundoscopy, biometric measurements (IOLMaster700, Carl Zeiss AG, Oberkochen, Germany), corneal topography (Pentacam HR, Oculus, Berlin, Germany), and B scans (Alcon Laboratories, Fort Worth, USA). The spherical and cylindrical IOL power and the axis of each toric IOL were calculated based on the anterior corneal astigmatism measured with IOLMaster, using the IOL manufacturer’s online calculator (version 1.5 containing the predicted posterior corneal astigmatism, https://zcalc.meditec.zeiss.com, accessed on 1 January 2023). The total corneal irregular astigmatism higher-order aberrations and coma aberrations in the 4 mm zone, measured with Pentacam, were also recorded for each eye.

### 2.3. Surgical Procedures

All the cataract surgeries were performed under topical anesthesia by one experienced surgeon. A 2.6 mm temporal clear corneal incision was created, followed by a 5.5 mm continuous curvilinear capsulorhexis, phacoemulsification, and the removal of the cortex. The toric IOL (CT Asphina 709MP, Carl Zeiss AG, Oberkochen, Germany) was implanted in the capsular bag under navigation with the Callisto Eye System (Carl Zeiss AG, Oberkochen, Germany). After the residual viscoelastics (DisCoVisc, Alcon Laboratories, Fort Worth, USA) were removed from above and below the IOL, the incision was hydrated, and the final IOL axis was checked and recorded. No stitches were used in any eye. The postoperative medications were the same in all patients.

### 2.4. Postoperative Follow-Up

Patients underwent follow-up examinations 3 months after surgery, including an assessment of visual acuity and manifest refraction at 5 m, corneal topography (Pentacam HR), and toric IOL axis alignment using a retroillumination photograph obtained with an OPD-Scan III corneal aberrometer (Nidek Co., Ltd., Gamagori, Japan) after mydriasis. The uncorrected distance and corrected distance visual acuities (in logarithm of the minimal angle of resolution), residual astigmatism, and misalignment of the IOL were recorded for each eye. The residual astigmatic refractive error, defined as the difference between the actual and predicted residual astigmatisms, was calculated and compared between the asymmetric and symmetric groups. The centroid and absolute mean values of the residual astigmatic refractive error were calculated in this study.

### 2.5. Actual Surgically Induced Astigmatism

The individual surgically induced astigmatism (SIA) of the cornea per eye was determined postoperatively using the vector difference between the postoperative and preoperative TCRP on Pentacam.

### 2.6. Analysis of the Accuracy of Corneal Astigmatism Measurements

To determine the accuracy of the measurements of corneal astigmatism in the asymmetric group, we analyzed the prediction errors (the differences between the estimated preoperative corneal astigmatism and the measured preoperative corneal astigmatism) obtained with IOLMaster and three Pentacam modes (SimK, TCRP, and WFA). The estimates of preoperative corneal astigmatism were calculated with the following equation, based on the previous studies performed by Eom et al. [20,21], as follows:

Estimated preoperative corneal astigmatism = postoperative residual astigmatism on the corneal plane (D_residual-cornea_) − toric IOL cylinder power on the corneal plane (D_IOL-cornea_) − actual surgically induced astigmatism of the cornea.

Postoperative residual astigmatism (D_residual_) and toric IOL cylinder power (D_IOL_) were first converted to the corneal plane with the following formulae:

$$D_{residual-cornea}=\frac{D_{residual}}{1-0.012\times D_{residual}}$$

$$D_{IOL-cornea}=\frac{1336}{\frac{1336}{\frac{1336}{\frac{1336\;}{K}-ELP}+D_{IOL}}+ELP}-K$$

The net corneal power (K) was measured with Pentacam and the effective lens position (ELP) was measured according to our previous study [22]. The alignment of the actual toric IOL axis, taken from the retroillumination photograph obtained postoperatively with OPD-Scan III, was used for the vector analysis. We then calculated the prediction errors with the following equation:

Prediction error = preoperative corneal astigmatism measured with each modality − estimated preoperative corneal astigmatism.

Both the centroid and absolute mean values for the prediction errors were calculated in this study.

### 2.7. Statistical Analysis

The vector analysis was obtained using an online calculator (www.isrs.org, accessed on 2 January 2023). All analyses were conducted with the SPSS software (version 23.0, IBM, San Francisco, CA, USA). Continuous data are presented as means ± standard deviations. Differences in continuous variables were compared between groups with Student’s *t* test, and differences in categorical variables were compared with a χ^2^ test. A paired *t* test was used to compare data before and after surgery within the same group. Multiple linear regression was used to analyze the factors influencing the absolute residual astigmatic refractive error. Friedman’s two-way analysis of variance by rank with a post hoc paired analysis was used to compare the absolute prediction errors in corneal astigmatism among the different measurement modes. *p* values of <0.05 were considered statistically significant.

## 3. Results

### 3.1. Baseline Characteristics

Table 1 shows the characteristics of both groups. In the asymmetric group, 13 eyes (43.3%) were with I–S > 1.5 D and the other 17 eyes (56.7%) were with S–I >2.5 D. The age, sex, operated eye, axial length, and corneal astigmatism determined with IOLMaster did not differ between the two groups (all *p* > 0.05; Student’s *t* test for age, axial length, and corneal astigmatism, and χ^2^ test for sex and operated eye). The difference in the distribution of corneal astigmatism orientations between the two groups was not significant (χ^2^ test, *p* > 0.05). However, the total irregular corneal astigmatism higher-order aberrations and coma aberrations were significantly greater in the asymmetric group than in the symmetric group (Student’s *t* test, both *p* < 0.05).

### 3.2. Postoperative Refractive Outcomes in the Two Groups

The postoperative misalignment of the toric IOL axis was similar in the two groups (4.33 ± 2.97° vs. 4.17 ± 2.74°, Student’s *t* test, *p* = 0.829). In the asymmetric and symmetric groups, the centroid mean values for the postoperative residual astigmatism were 0.40 D@176° and 0.28 D@174°, respectively, and the absolute mean values were 0.69 ± 0.41 D and 0.52 ± 0.30 D, respectively (Student’s *t* test, *p* = 0.066), both of which were significantly lower than the preoperative corneal astigmatism (paired *t* test, both *p* < 0.05). The residual astigmatism was more than 0.5 D in 47% (14/30) of eyes in the asymmetric group and in 30% (9/30) of eyes in the symmetric group (χ^2^ test, *p* = 0.184). Among the eyes with more than 0.5 D of residual astigmatism, nine were overcorrected in the asymmetric group, versus one eye in the symmetric group (Fisher’s exact test, *p* = 0.029).

Figure 2 shows the double-angle plots of the residual astigmatic refractive errors in both groups. The centroid residual astigmatic refractive errors were 0.53 D@175° in the asymmetric group and 0.31D@173° in the symmetric groups. The mean absolute residual astigmatic refractive error was significantly greater in the asymmetric group than in the symmetric group (0.72 ± 0.42 D vs. 0.53 ± 0.24 D, respectively, Student’s *t* test, *p* = 0.043). The asymmetric group had more eyes with residual astigmatic refractive errors more than 1 D compared with the symmetric group (Table 2). After adjustment for age, sex, operated eye, axial length, corneal astigmatism value, and misalignment of the toric IOL axis, we found that asymmetric bowtie astigmatism was a significant risk factor (relative to symmetric bowtie astigmatism) for the absolute residual astigmatic refractive error (multiple linear regression, β = 0.309, *p* = 0.021).

### 3.3. Corneal Astigmatism Prediction Errors in Asymmetric Group

The double-angle plots of the prediction errors measured using the four different modes in the asymmetric group are shown in Figure 3A–D. The centroid prediction errors obtained with the IOLMaster, SimK, TCRP, and WFA modes were 0.23 D@80°, 0.25 D@80°, 0.26 D@78°, and 0.10 D@88°, respectively. The mean absolute prediction errors of the four modes were 0.53 ± 0.40, 0.56 ± 0.47, 0.68 ± 0.52, and 0.43 ± 0.40 D, respectively, and the values differed significantly among all four modes (Friedman’s two-way analysis of variance, *p* = 0.002). A post-hoc paired test showed that the WFA mode had a significantly lower absolute prediction error than the TCRP mode (*p* = 0.002). However, there were no significant differences between the other modes.

The proportions of eyes with an absolute prediction error of ≤0.5 D or ≤1.0 D according to the measurement mode are shown in Figure 4. The proportions were the greatest in the WFA mode, with an absolute prediction error of ≤0.5 D for 83.3% of eyes and ≤1.0 D for 90% of eyes.

The absolute prediction error of the WFA mode was also positively correlated with the total irregular astigmatism higher-order aberration and the coma aberration of the cornea (Pearson’s r = 0.416 and r = 0.473, both *p* < 0.05; Figure 5).

## 4. Discussion

Previous studies have demonstrated the good performance of toric IOLs in eyes with regular symmetric corneal astigmatism following improvements in biometric accuracy [4]. However, many surgeons remain cautious about the use of toric IOLs for correcting asymmetric bowtie astigmatism because there is no ideal surgical plan. Nevertheless, a viewpoint has recently been reached that even partial correction of corneal astigmatism during cataract surgery might benefit these patients, as long as it is properly planned [10]. Therefore, to provide evidence supporting this viewpoint, we first evaluated the refractive outcomes of toric IOL implantation in eyes with asymmetric bowtie astigmatism, and then compared the accuracy of different measurement modes within this context. We found that, although the residual astigmatic refractive error was greater in the asymmetric group than in the symmetric group, the postoperative residual astigmatism of the asymmetric group was significantly improved relative to the preoperative state. For these eyes, the Pentacam WFA mode may be the preferable measurement mode. However, its accuracy decreased as the degree of asymmetry increased.

Our data show that toric IOLs can be effectively used to correct asymmetric bowtie corneal astigmatism. Many studies have examined the efficacy of toric IOL implantation during cataract surgery. A systematic review and meta-analysis showed that the average residual astigmatism at 3–6 months after toric IOL implantation was 0.53 D, ranging from 0.18 to 0.77 D [4], which is similar to our results for the symmetric group. Cases referred to as ‘asymmetric astigmatism’ in previous studies often had keratoconus, pterygium, or history of corneal surgery, for example [10,23,24,25], which are typically regarded as irregular astigmatism and cannot be completely corrected. The mean residual astigmatism in these cases was 0.87 ± 1.10 D, ranging from 0 to 5.50 D, which is higher than our result for the asymmetric group. This discrepancy arises because we focused on a specific type of asymmetric astigmatism, asymmetric bowtie astigmatism, in which the power on both sides differs, although the orientations are same. Therefore, there are symmetric components in this type of asymmetric astigmatism. We investigated whether the astigmatism in these patients could be corrected with toric IOLs. We found that the postoperative residual astigmatism was significantly reduced, from 2.18 ± 0.70 D to 0.69 ± 0.41 D, confirming that cataract surgery with toric IOL implantation is effective in correcting asymmetric bowtie astigmatism, with better results than those reported for irregular astigmatism in previous studies [10,23,24,25]. However, irregular astigmatism may have more irregular aberrations that cannot be corrected by toric IOLs and lead to a greater residual astigmatism.

As expected, the error between the actual and predicted postoperative residual astigmatism was greater in the asymmetric group, and overcorrection was more frequent in these eyes. A possible explanation is that, if the power of the toric IOL was simply calculated based on the available biometric data, which are largely affected by the power of the larger semi-bowtie, the astigmatism may be overestimated.

A lot of work has been carried out to reduce residual refractive error after cataract surgery. Accurate assessment of the ocular biometrics plays an important role in these efforts. Many studies compared the accuracy of different methods to measure astigmatism. A study compared the keratometry measurements obtained from IOLMaster and Pentacam and showed that IOLMaster had superior performance in prediction of postoperative astigmatism, which is different from ours [26]. Another study aimed to compare the effect of corneal irregularity on astigmatism assessment using IOLMaster and Pentacam. They found corneal irregularities could significantly impact astigmatism assessment by both instruments and Pentacam (TCRPs) was more accurate in predicting postoperative residual astigmatism in highly irregular corneas [27]. Tana-Rivero et al. compared the ocular parameters using three different devices (ANTERION, IOL Master 700, and Pentacam) and found no significant difference in Ks and Kf [28]. The difference may due to difference of the patients, devices, and ocular conditions. Interestingly, we found that among the four measurement modes tested, the Pentacam WFA mode showed the greatest accuracy for asymmetric bowtie. Asymmetric bowtie astigmatism can be seen as a combination of symmetric bowtie and coma, and only the former can be corrected by a toric IOL. The Hartman–Shack aberrometer is used in Pentacam to capture wavefront data at multiple locations [29]. The Hartman–Shack aberrometer has better repeatability than other sensor technologies [30]. Pentacam WFA measures the regular part of the corneal astigmatism based on the second-order aberration of the astigmatism of the total cornea, whereas other measurement modes, especially the Pentacam TCRP mode, include the coma aberration of the larger semi-bowtie, which may overestimate the magnitude of the corneal astigmatism.

We also found that, as the corneal asymmetry increased, the accuracy of the WFA mode in calculating the toric IOL decreased. Based on a computational model, previous studies have estimated that the refractive effect of a root-mean-square higher-order aberration of ≥0.43 μm is equivalent to a spherical error of ≥0.5 D for a 5 mm pupil aperture [31,32]. Another study showed that the vertical and horizontal coma of the cornea influence the refractive astigmatism in different ways [33]. Therefore, in cases with a higher-order aberration of the cornea of ≥0.43 μm or in corneas with large coma aberrations, their effects should be taken into consideration in the calculation of toric IOL.

There are also some limitations in our study. Firstly, our research is a retrospective, single-center study. Multicenter, large sample, and prospective clinical studies are needed to further confirm the results. Additionally, the effect of astigmatism axis position and bowtie size should be analyzed using a large cohort. Secondly, total corneal keratometry could also be measured by IOLMaster 700. It may provide more accurate measurement for asymmetric bowtie corneal astigmatism and further studies need to be conducted in this regard.

In conclusion, toric IOL can be used to correct asymmetric bowtie corneal astigmatism. Corneal astigmatism measured with the Pentacam WFA mode may be a better choice for surgical planning in these cases. However, its accuracy may decrease as the corneal asymmetry increases.

## Figures and Tables

**Figure 1:**
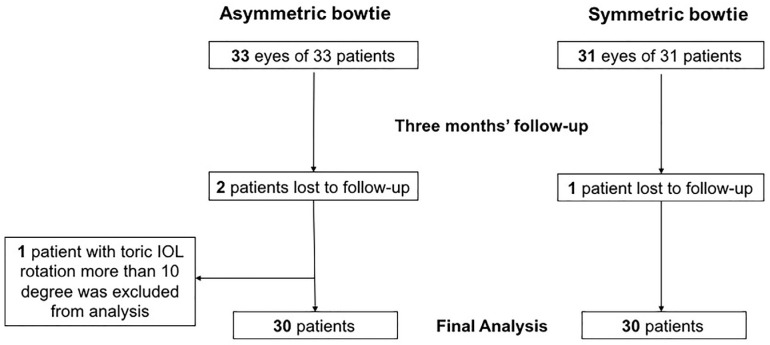
Patient flow chart.

**Figure 2:**
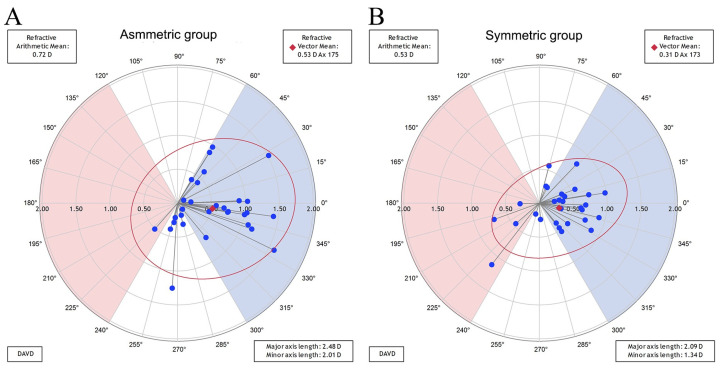
Double-angle plots of residual astigmatic refractive errors in the asymmetric and symmetric bowtie astigmatism groups. (**A**) asymmetric bowtie astigmatism group; (**B**) symmetric bowtie astigmatism group; The centroid (red plot) is the arithmetic mean of astigmatism of individual cases (blue plot). The centroid residual astigmatic refractive errors were 0.53 D@175° and 0.31 D@173° in the asymmetric and symmetric groups, respectively. The asymmetric group included more eyes with a residual astigmatic refractive error of >1.0 D.

**Figure 3:**
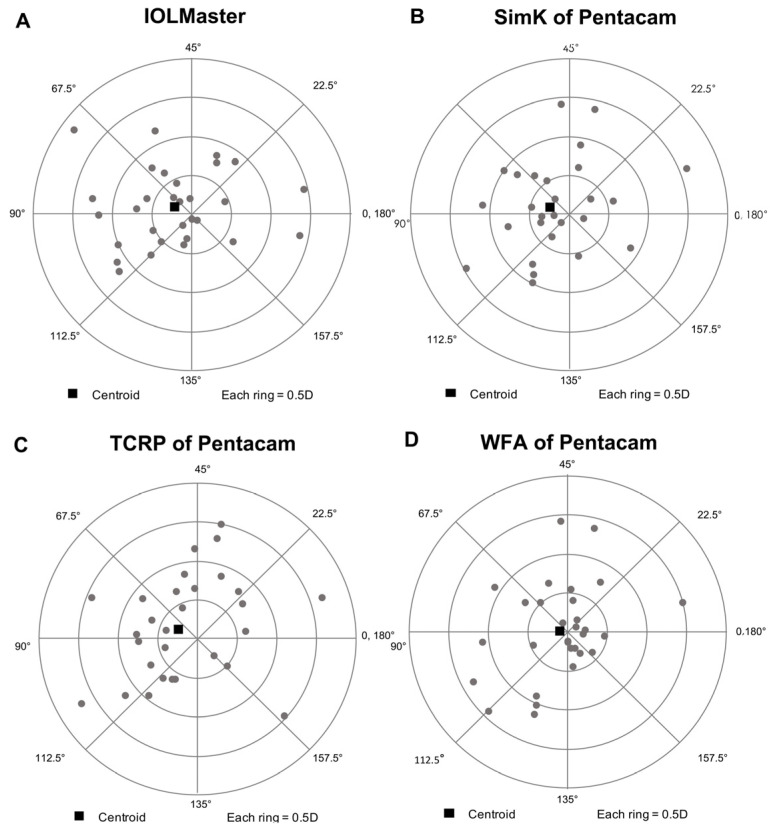
Double-angle plots of corneal astigmatism prediction errors in eyes, measured with the four different measurement modes: (**A**). IOLMaster; (**B**). Pentacam SimK mode; (**C**). Pentacam TCRP mode; (**D**). Pentacam WFA mode. The centroid prediction errors of the four modes were 0.23 D@80°, 0.25 D@80°, 0.26 D@78°, and 0.10 D@88°, respectively. SimK, simulated keratometry; TCRP, total corneal refractive power; WFA, wavefront aberration.

**Figure 4:**
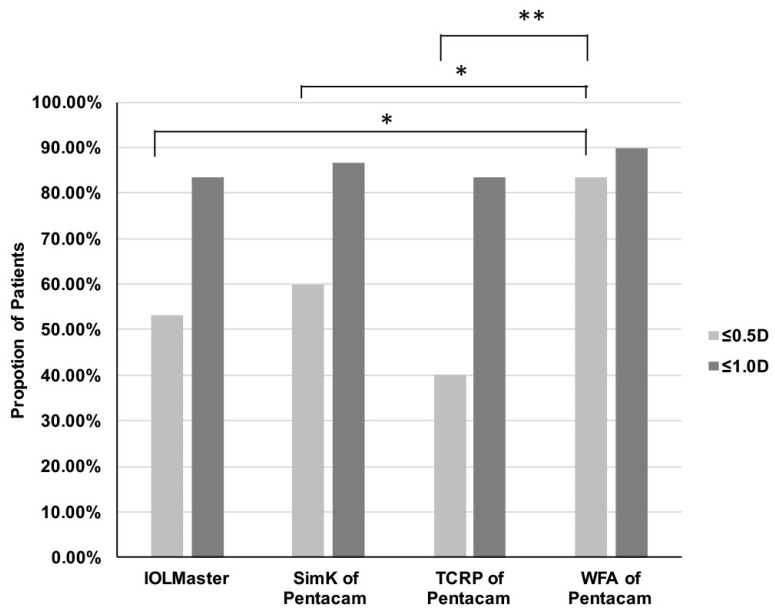
Proportion of eyes with absolute corneal astigmatism prediction errors of ≤0.5 D or ≤1.0 D, according to the measurement mode. The proportions were highest using the Pentacam WFA mode. The proportions of eyes with a prediction error of ≤0.5 D were significantly different (χ^2^ test, *p* = 0.007; WFA vs. IOLMaster, *p* = 0.012; WFA vs. SimK, *p* = 0.045; WFA vs. TCRP, *p* = 0.001). SimK, simulated keratometry; TCRP, total corneal refractive power; WFA, wavefront aberration. * *p* < 0.05, ** *p* < 0.01.

**Figure 5:**
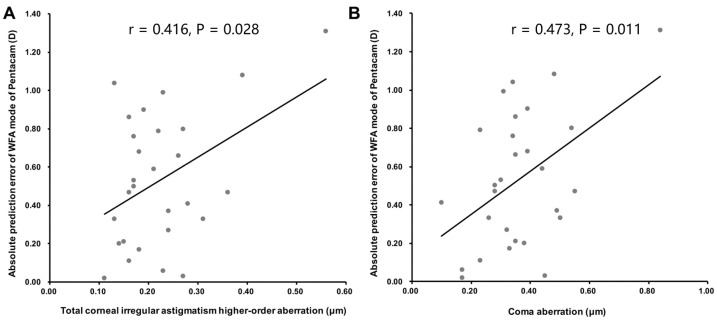
Correlations between the absolute prediction error determined using the WFA mode and corneal asymmetry. The absolute prediction error of the WFA mode was positively correlated with the total corneal irregular astigmatism higher-order aberration ((**A**); Pearson’s r = 0.416, *p* = 0.028) and with the coma aberration ((**B**); Pearson’s r = 0.473, *p* = 0.011).

**Table 1:** Characteristics of patients in the asymmetric and symmetric bowtie corneal astigmatism groups.

Parameters	Asymmetric Group	Symmetric Group	*p* Value
Age (year)	63.33 ± 12.78 (26–86)	60.53 ± 15.72 (39–83)	0.452
Sex (Male/Female)	7/23	12/18	0.171
Operated Eye (Right/Left)	16/14	15/15	0.800
Axial length (mm)	26.78 ± 3.20 (22.27–33.37)	26.20 ± 2.85 (21.94–34.6)	0.464
Anterior corneal astigmatism from IOLMaster (D)	2.18 ± 0.70 (1.09–3.88)	2.02 ± 0.78 (1.17–3.55)	0.398
Total corneal irregular astigmatism higher-order aberration (RMS, μm)	0.22 ± 0.09 (0.07–0.38)	0.16 ± 0.07 (0.11–0.56)	0.004
Coma aberration (RMS, μm)	0.37 ± 0.15 (0.02–0.53)	0.17 ± 0.10 (0.10–0.84)	<0.001

D = diopter; RMS = root mean square.

**Table 2:** Comparison of the distribution of residual astigmatic refractive errors between asymmetric and symmetric bowtie corneal astigmatism groups.

Distribution	Asymmetric Group *n* (%)	Symmetric Group *n* (%)	*p* Value
<0.5 D	11 (36.7)	15 (50)	0.297
0.5–1.0 D	10 (33.3)	14 (46.7)	0.292
1.0–1.5 D	8 (26.7)	1 (3.3)	0.026
>1.5 D	2 (6.7)	0 (0)	0.492

## Data Availability

The dataset generated during and/or analyzed during the currentstudy are available from the corresponding author on reasonable request.
